# Eosinophilic Meningitis and Intraocular Infection Caused by *Dirofilaria* sp. Genotype Hongkong

**DOI:** 10.3201/eid2705.203599

**Published:** 2021-05

**Authors:** Aruna S. Jyotsna, Kollencheri Puthenveettil Vinayan, Lalitha Biswas, Sujithra Haridas, Arun G. Roy, Parasmal Suresh, Anil Kumar

**Affiliations:** Amrita Institute of Medical Sciences, Amrita Viswa Vidyapeetham Cochin, Iindia

**Keywords:** dirofilariasis, eosinophilic meningitis, meningitis/encephalitis, ocular parasite, intraocular infection, Dirofilaria, genotype Hongkong, parasites, roundworms, nematodes, filarial nematodes

## Abstract

Eosinophilic meningitis caused by human diroflarial infection is rare. We report a case of eosinophilic meningitis and concomitant intraocular dirofilarial infection in India. Sequencing of the mitochondrial genome identified the worm as *Dirofilaria* sp. genotype Hongkong, a close relative of *D. repens* nematodes.

Dirofilariasis is a group of mosquitoborne parasitoses. The most prevalent *Dirofilaria* species causing infection are *D. imitis* and *D. repens* nematodes (*1*). Dogs are the definitive hosts in the life cycle, in which microfilaremia is observed. Humans are aberrant hosts, and the worms usually remain infertile (*1*,*2*). Human dirofilariasis is reported mostly as 1 worm in the subconjunctival or subcutaneous spaces. Surgical extraction of the worm constitutes definitive therapy. These worms are rarely observed inside the eye (*1*,*2*). Identification of the worm by using morphologic features is difficult because a large number of *Dirofilaria* species have similar features.

Diagnosis of eosinophilic meningitis is based mainly on clinical features and microscopic identification of eosinophils in the central nervous system. Helminthic infections, such as angiostrongylosis, baylisascariasis, and gnathostomiasis, are most commonly implicated in eosinophilic meningitis (*3*). We report a rare case of eosinophilic meningitis and concomitant intraocular dirofilarial infection. Sequencing of the mitochondrial genome of the extracted worm identified it as *Dirofilaria* sp. genotype Hongkong, a close relative of *D. repens* (*4*).

A 17-year-old woman came to our institute in Kochi, India, because of acute onset of severe headache, irritability, visual blurring, and diplopia, after 3 weeks of intermittent fever. She had meningeal signs, bilateral lateral rectus palsy, and papilledema. Peripheral eosinophilia (14.2%) was observed. Magnetic resonance imaging of the brain (Appendix Figure 1) showed diffuse leptomeningeal enhancement. Cerebrospinal fluid showed lymphocytic pleocytosis (1,040 cells/μL), major eosinophilia (37%), and protein and glucose levels within reference ranges.

A live worm was detected in the anterior chamber of her left eye (Figure, panel A), confirmed by slit lamp examination (Figure, panel B; Video). The lens showed cataractous changes. Indirect ophthalmoscopy showed inflammatory changes in retinal pigment epithelium, suggestive of a migratory tract. Serologic analysis for helminthic antibodies was not conducted because serologic testing was were not available. A white, thread-like worm (length ≈15 mm) was extracted after the worm was paralyzed by injection of lignocaine into the anterior chamber of the eye (Figure, panel C).

**Figure:**
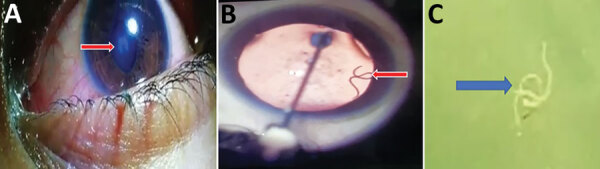
Eosinophilic meningitis and intraocular infection caused by *Dirofilaria* sp. genotype Hongkong in a patient in Kochi, Inida. A) Organism (arrow) in the left eye of patient during routine clinical examination. The organism caused an abnormal shape of the pupil. B) Live worm (arrow) in anterior chamber of the left eye. This image was obtained while lignocaine was being injected. C) Gross specimen of the worm (arrow) after extraction. Worm is in saline in a Petri dish.

**Video:** Live, mobile worm in anterior chamber of left eye of the patient. Video was taken while lignocaine was being injected.

Because a PCR was available, histopathologic analysis was not conducted. Morphologic features or sex could not be determined. The worm specimen was subjected to multiplex PCR for *D. repens* and *D. imitis* using an equimolar combination of general and species-specific primers: Diro_12S_F (5′-GTTCCAGAATAATCGGCTA-3′), Diro_12S_R (5′-ATTGACGGATGGTTTGTACC-3′), *D. immitis*_F (5′-TTTTTACTTTTTTGGTAATG-3′), and *D. repens*_R (5′-AAAAGCAACACAAATAAAA-3′). The cytochrome c oxidase subunit 1 (COX1) region was amplified by using primers Fil_COX1F (5′-GCTTTRTCTTTTTGGKTTACTTTT-3′) and Fil_COX1R (5′-TAGTRTCATAAAAAGAAGTATTAAA-3′) (*5*).

Although the specimen was identified as a *D. repens* worm, Sanger sequencing of the COX1 and 12S rDNA PCR products was performed by using the BigDye Terminator v3.1 Cycle Sequencing Kit (Applied Biosystems, https://www.thermofisher.com) and the Genetic Analyzer 3130XL (Applied Biosystems). Sequences of 12S rRNA and COXI genes obtained were deposited in GenBank (accession nos. MT984272 and MT984209).

Phylogenetic analysis of the 12S rRNA (MT984272) and COX1 (MT984209) sequences obtained from the isolate was performed by using the maximum-likelihood method with 1,000 bootstrap replications and MEGA X version 7 (https://www.megasoftware.net). Both the 12S rRNA and the COX1 sequences obtained from the human isolate were in the same cluster with *Dirofilaria* sp. genotype Hongkong and were separated from other *Dirofilaria* species (*5*,*6*) (Appendix Figure 2). Peripheral blood smears were negative for microfilaria. Symptoms of the patient resolved slowly after worm extraction and initiation of treatment with steroids.

Migrating worms in humans might cause a variety of clinical problems, which could be caused by mechanical effects or immune responses. Intraocular parasites might induce severe damage to various structures in the eye. Literature on eosinophilic meningitis and concomitant ocular parasites is limited. Clinical manifestations of eosinophilic meningitis are usually attributed to the severe inflammatory response incited by migrating worms, even though they are rarely demonstrated in vivo. Eosinophilic meningitis caused by *Angiostrongylus cantonensis* worms has been frequently reported in the Asia–Pacific region (*7*). *Dirofilaria* infection rarely results in eosinophilic meningitis (*1*,*2*).

Poppert et al. reported a case of *D. repens* infection, which was subsequently identified as *Dirofilaria* sp. genotype Hongkong, which caused subcutaneous infection and concomitant eosinophilic meningoencephalitis in a traveler returning from Kerala, India, and Sri Lanka to Germany (*8*). Subconjunctival infection with *Dirofilaria* sp. genotype Hongkong has also been reported in a patient returning to Austria after a 7-week stay in India (*9*). A recent study from Kerala, India, suggested that most of *D. repens* infections reported from southern India have the *Dirofilaria* sp. Hongkong genotype (*10*).

Demonstration of a live, intraocular worm and its subsequent identification as *Dirofilaria* sp. genotype Hongkong by using sequencing added a new dimension to this case of eosinophilic meningitis. Infection with the *Dirofilaria* sp. Hongkong genotype, blood eosinophilia, and eosinophilic meningitis are the 3 strikingly similar features between our case-patient and Poppert et al. (*8*), suggesting that *Dirofilaria* sp. genotype Hongkong might induce a more systemic eosinophilic reaction than *D. repens.*

Sequencing using panfilarial primers might help characterize most filarial species. Such an approach might clarify the etiopathogenesis of eosinophilic meningitis, leading to newer therapeutic and preventive strategies.

Appendix Additional information on eosinophilic meningitis and intraocular infection caused by *Dirofilaria* sp. genotype Hongkong.
