# Supplementary figures and images for: Eosinophilic Meningitis and Intraocular Infection Caused by Dirofilaria sp. Genotype Hongkong
Source: Emerg Infect Dis. 2021 May;27(5):1532–4. doi: 10.3201/eid2705.203599 (PMC8084481; doi:10.3201/eid2705.203599)

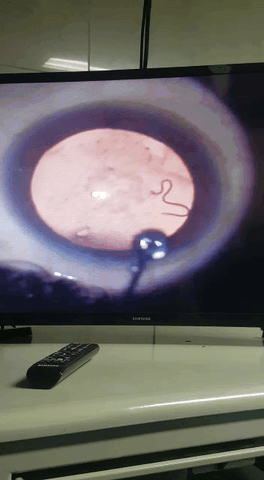

Supplement: Supplementary file 1 [file 20-3599-vid1.gif]
