# Supplementary material for: Eosinophilic Meningitis and Intraocular Infection Caused by Dirofilaria sp. Genotype Hongkong
Source: Emerg Infect Dis. 2021 May;27(5):1532–4. doi: 10.3201/eid2705.203599 (PMC8084481; doi:10.3201/eid2705.203599)
Supplement: Appendix — Additional information on eosinophilic meningitis and intraocular infection caused by Dirofilaria sp. genotype Hongkong. [file 20-3599-Techapp-s1.pdf]

Article DOI: https://doi.org/10.3201/eid2705.203599

Eosinophilic Meningitis and Intraocular Infection Caused by *Dirofilaria* sp. Genotype Hongkong

Appendix

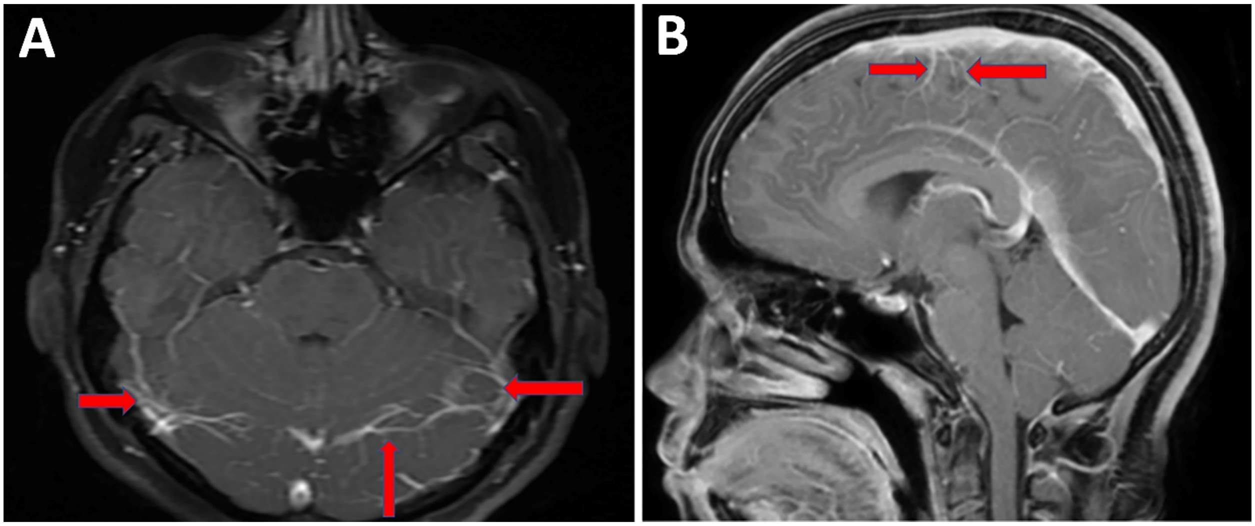

Appendix Figure 1. Magnetic resonance imaging of brain of patient infected with *Dirofilaria* sp. genotype Hongkong. A) Post contrast, axial T1 image and B) sagittal image show diffuse leptomeningeal enhancement.

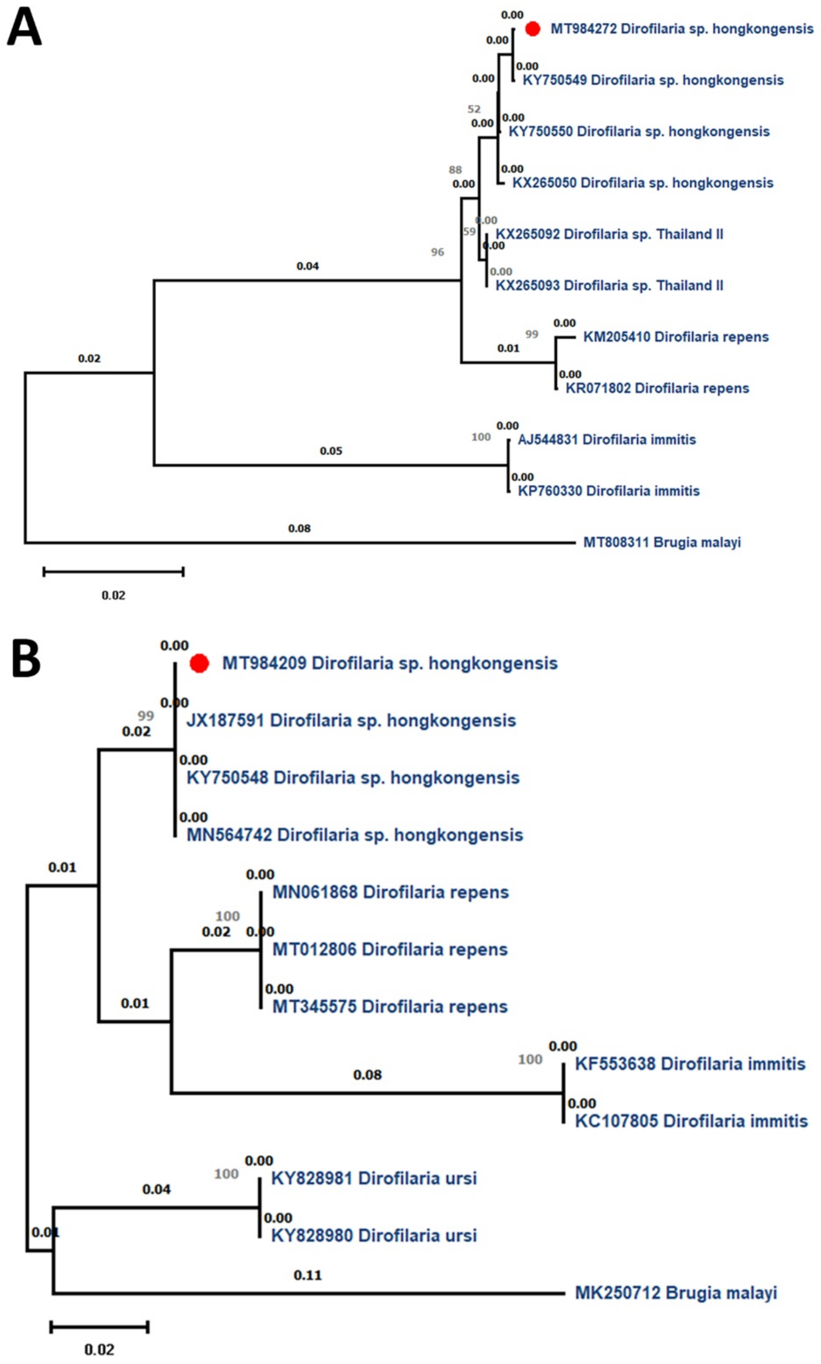

Appendix Figure 2. Phylogenetic analysis of *Dirofilaria* sp. genotype Hongkong worm isolated from the patient. A) 12S rRNA gene sequences of the *Dirofilaria* species. *Dirofilaria* sp. genotype Hongkong. B) Phylogenetic tree of cytochrome c oxidase subunit 1 gene sequences of the *Dirofilaria* species. *Brugia malayi* was used as the outgroup. Numbers along branches are bootstrap values. Red dots indicate *Dirofilaria* isolated in the present study. Scale bars indicate nucleotide substitutions per site.
